# Role of Cutaneous Aquaporins in the Development of Xeroderma in Type 2 Diabetes

**DOI:** 10.3390/biomedicines9020104

**Published:** 2021-01-21

**Authors:** Nobutomo Ikarashi, Nanaho Mizukami, Chenchen Pei, Ryogo Uchino, Izumi Fujisawa, Natsuko Fukuda, Risako Kon, Hiroyasu Sakai, Junzo Kamei

**Affiliations:** Department of Biomolecular Pharmacology, Hoshi University, 2-4-41 Ebara, Shinagawaku, Tokyo 142-8501, Japan; cffcx065@gmail.com (N.M.); pei_chenchen@163.com (C.P.); swi10m19r_3102.star@mineo.jp (R.U.); aqua07wing_sky48@i.softbank.jp (I.F.); s151206@hoshi.ac.jp (N.F.); r-kon@hoshi.ac.jp (R.K.); sakai@hoshi.ac.jp (H.S.)

**Keywords:** diabetes, aquaporin, skin, xeroderma, inflammation, TNF-α, COX-2, iNOS

## Abstract

Xeroderma is induced by diabetes, reducing patients’ quality of life. We aimed to clarify the roles of cutaneous water channel aquaporin-3 (AQP3) in diabetic xeroderma using type 2 diabetes model *db/db* mice. Blood glucose levels were unchanged in 5-week-old *db/db* mice compared to *db/+* mice (control mice), but the pathophysiology of type 2 diabetes was confirmed in 12-week-old *db/db* mice. The dermal water content and AQP3 expression in 5-week-old *db/db* mice were almost the same as those in the control mice. On the other hand, in 12-week-old *db/db* mice, the dermal water content and AQP3 expression were significantly decreased. The addition of glucose to HaCaT cells had no effect on AQP3, but tumor necrosis factor-α (TNF-α) decreased the AQP3 expression level. Blood TNF-α levels or skin inflammation markers in the 12-week-old *db/db* mice were significantly higher than those in control mice. AQP3 levels in the skin were decreased in type 2 diabetes, and this decrease in AQP3 may be one of the causes of xeroderma. Therefore, a substance that increases AQP3 may be useful for improving xeroderma. Additionally, a decrease in skin AQP3 may be triggered by inflammation. Therefore, anti-inflammatory drugs may be effective as new therapeutic agents for diabetic xerosis.

## 1. Introduction

The number of patients with diabetics is increasing worldwide due to changes in lifestyle and social factors [1]. Polyuria and thirst occur as initial symptoms at the beginning of diabetes, after which nephropathy, retinopathy, and peripheral neuropathy develop. In clinical practice, many diabetic patients also develop skin diseases, such as skin infection, xerosis, and inflammatory skin diseases [2,3,4]. Among them, the most common type of skin infection, found in approximately half of diabetic patients, is mainly caused by the destruction of immunity [5,6]. Since skin infections are completely cured by the proper use of antibacterial drugs, they rarely pose a major clinical problem. On the other hand, xeroderma, a skin disease characterized by dryness of the skin due to a decrease in the dermal water content, occurs in one of four diabetic patients [2,3,4]. Since xeroderma is not a life-threatening disease in patients, its treatment is often neglected. However, xerosis induces itch and decreases patients’ quality of life. In addition, since xeroderma can cause other skin diseases, such as skin infections and skin gangrene, its prevention and treatment are very important. Currently, moisturizers are used as a symptomatic treatment for xeroderma. Moisturizers are effective when applied many times a day, but their therapeutic effect is not sufficient. Therefore, many scientists aim to clarify the onset mechanism of this disease to prevent and treat diabetic xerosis, but the entire process has not yet been elucidated.

Aquaporins (AQPs) were recently shown to play an important role in water transport in the body. AQP is a water channel in the cell membrane that transports water through cells based on the osmotic pressure gradient. Among AQPs, 13 subfamilies from AQP0 to AQP12 are widely distributed throughout the human body. In particular, AQP3 is highly expressed in the skin, and in AQP3-knockout mice, the dermal water content and flexibility and elasticity of the skin were reduced [7]. We previously analyzed the role of cutaneous AQP3 in the development of diabetic xerosis using streptozotocin (STZ)-induced type 1 diabetes model mice [8]. As a result of modeling, blood glucose levels were increased in STZ-treated mice, and the dermal water content and cutaneous AQP3 expression were decreased. The results showed that the expression level of AQP3 in the skin was reduced in type 1 diabetes, which may be one of the factors that cause xeroderma. However, the association between xeroderma and AQP3 expression in type 2 diabetes, which accounts for the majority of diabetes cases, remains unclear. Therefore, in this study, we clarified the overall role of cutaneous AQP3 in the development of diabetic xerosis using type 2 diabetic model mice to investigate new preventive and therapeutic methods targeting AQP3. In particular, we used *db/db* mice as a model of type 2 diabetes to examine changes in the dermal water content and cutaneous AQP3 expression with the development of diabetes. Furthermore, we attempted to elucidate the mechanism by which the expression level of AQP3 in the skin changed. In the mechanism analysis, we focused on inflammation, especially tumor necrosis factor-α (TNF-α), based on the report that inflammation is induced in type 2 diabetes patients [9] and that TNF-α regulates the expression of skin AQP3 [10].

## 2. Experimental Section

### 2.1. Materials

A glucose CII-test kit was purchased from Fujifilm Wako Chemicals Co., Ltd. (Osaka, Japan). A mouse insulin enzyme-linked immunosorbent assay (ELISA) kit was purchased from Shibayagi Co., Ltd. (Gunma, Japan). A mouse tumor necrosis factor-α (TNF-α) Quantikine ELISA kit was purchased from R&D Systems (Minneapolis, MN, USA). RIPA buffer was purchased from Nakarai Tesque (Kyoto, Japan). TRI reagent was purchased from Sigma–Aldrich Corp. (St. Louis, MO, USA). A high-capacity cDNA synthesis kit was purchased from Applied Biosystems (Foster City, CA, USA). SsoAdvanced Universal SYBR Green Supermix was purchased from Bio-Rad Laboratories (Hercules, CA, USA). Rabbit anti-rat AQP3 antibody was purchased from Alomone Labs (Jerusalem, Israel). Mouse anti-rabbit glyceraldehyde-3-phosphate dehydrogenase (GAPDH) antibody was purchased from Chemicon International, Inc. (Temecula, CA, USA). Donkey anti-rabbit immunoglobulin G (IgG)-horseradish peroxidase (HRP) antibody was purchased from Santa Cruz Biotechnology, Inc. (Santa Cruz, CA, USA). Donkey anti-mouse IgG-HRP antibody and enhanced chemiluminescence (ECL) Prime western blotting detection reagent was purchased from GE Healthcare (Chalfont St. Giles, UK).

### 2.2. Animals

Five-week-old male *db/db* mice and *db/+* mice (control mice) were purchased from Japan SLC, Inc. (Shizuoka, Japan) and used for experiments at 5 and 12 weeks of age. The animals were housed at 24 ± 1 °C and 55 ± 5% humidity under light from 8:00–20:00. Food (MF diet used for experimental animals, Oriental Yeast Co., Ltd., Tokyo, Japan) and water were available. In this study, we followed the guidelines for animal experiments from Hoshi University and obtained approval (approval number: 19-058) from the Animal Experiment Committee at the university.

### 2.3. Treatment

The backs of the mice were shaved, and the dermal water content was measured with a Tewameter (TM300, Courage & Khazaka, Cologne, Germany). Under nonfasting conditions, blood was collected under isoflurane anesthesia, and the skin (back) was removed. The skin was instantly frozen in liquid nitrogen and then stored at −80 °C. The dermal water content was measured at a temperature of 23 ± 1 °C and 60 ± 10% humidity.

### 2.4. Measurements of Blood Glucose, Insulin, and TNF-α Levels

The collected blood was centrifuged (1000× *g* for 15 min at 4 °C) to separate the plasma. Plasma glucose, plasma insulin, and TNF-α concentrations were measured with a glucose CII test kit, mouse insulin ELISA kit, and mouse TNF-α Quantikine ELISA kit, respectively.

### 2.5. Cell Culture

HaCaT cells (Cell Line Service, Eppelheim, Germany) were maintained in Dulbecco’s modified Eagle medium (DMEM) containing 100 U/mL penicillin G potassium, 100 μg/mL streptomycin, and 10% fetal bovine serum.

### 2.6. Glucose Treatment of HaCaT Cells

The cells were plated on a culture plate and maintained in DMEM (glucose concentration: 1000 mg/L). After the cells reached subconfluence, the medium was replaced with DMEM containing glucose at 1000 mg/L or 4500 mg/L, and the cells were incubated for 24 h.

### 2.7. TNF-α Treatment of HaCaT Cells

The cells were plated on a culture plate and maintained in DMEM (glucose concentration: 4500 mg/L). TNF-α (1–10 μM) was added to the subconfluent cells, which were then cultured for 24 h. Ethanol (final concentration: 0.1%) was added to the control cells.

### 2.8. Real-Time RT-PCR

Total RNA was extracted from mouse skin or HaCaT cells using TRI reagent. cDNA was synthesized from 1 µg of RNA using a high-capacity cDNA synthesis kit. The various primers shown in Supplementary Table S1 were prepared, and real-time PCR was performed to detect the expression of each gene. Briefly, SsoAdvance Universal SYBR Green Supermix, forward primer/reverse primers for the target gene, and a cDNA solution were added to each well of a PCR plate. The fluorescence intensity was monitored with a CFX Connect real-time PCR detection system (Bio-Rad Laboratories). mRNA expression levels were normalized using mβ-actin or hGAPDH.

### 2.9. Preparation of Mouse Skin Samples for Western Blotting

The skin was homogenized on ice with dissection buffer (0.3 M sucrose, 25 mM imidazole, 1 mM ethylenediaminetetraacetic acid, 8.5 μM leupeptin, and 1 μM phenylmethylsulfonyl fluoride; pH 7.2) using a homogenizer. The homogenate was dispersed by an ultrasonic homogenizer and centrifuged (4000× *g* for 15 min at 4 °C). The obtained supernatant was centrifuged (9000× *g* for 10 min at 4 °C), after which the supernatant was centrifuged (200,000× *g* for 60 min at 4 °C). The supernatant was removed, and dissection buffer was added to the precipitate. This solution was used as a sample solution for western blotting [11].

### 2.10. Preparation of HaCaT Cell Samples for Western Blotting

RIPA buffer was added to HaCaT cells, and the cells were collected with a cell scraper. After homogenization on ice, the homogenate was dispersed with an ultrasonic disperser and centrifuged (15,000× *g* for 15 min at 4 °C). The obtained supernatant was used as a sample solution for western blotting [12].

### 2.11. Western Blotting

The protein concentration was measured by the bicinchoninic acid (BCA) method with bovine serum albumin (BSA) as a standard. Loading buffer (100 mM Tris, 20% glycerol, 0.004% bromophenol blue, 4% sodium dodecyl sulfate, and 10% 2-mercaptoethanol; pH 6.8) was added to the sample solution, and electrophoresis was performed on a polyacrylamide gel. The protein was transferred to a polyvinylidene difluoride membrane, and the membrane was blocked with a skim milk solution. The membrane was reacted with a primary antibody solution at room temperature. After washing, the membrane was reacted with a secondary antibody solution at room temperature. After washing, the membrane was then reacted with ECL Prime western blotting detection reagent. The membrane was photographed with a CCD camera (ImageQuant LAS500, GE Healthcare), and the band density was analyzed.

### 2.12. Statistical Analysis

The experimental values are shown as the mean ± standard deviation (S.D.) Student’s *t*-test and one-way ANOVA followed by Dunnett’s test were used to assess the statistical significance of differences.

## 3. Results

### 3.1. Body Weight, Blood Glucose Level, and Blood Insulin Level

**Figure 1:**
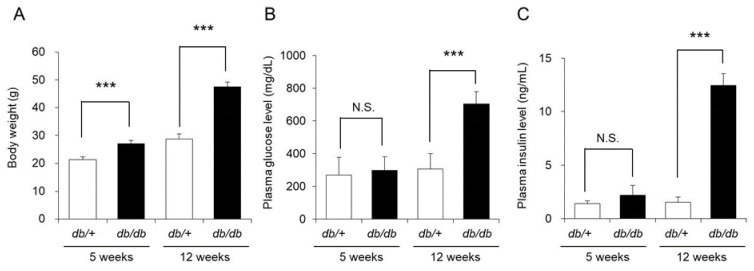
Body weight, blood glucose level, and blood insulin level. The body weight (**A**), blood glucose level (**B**), and blood insulin level (**C**) of *db/+* mice and *db/db* mice at 5 and 12 weeks of age were measured (mean ± SD, *n* = 5, N.S.: not significant, ***: *p* < 0.001).

The 5-week-old *db/db* mice weighed significantly more than the *db/+* mice (control mice). However, the blood glucose and blood insulin levels in the two groups were almost the same. The 12-week-old *db/db* mice weighed approximately 48 g, which was significantly higher than the weight of the control mice (approximately 29 g). The blood glucose and blood insulin levels of the 12-week-old *db/db* mice were also significantly higher by approximately 2-fold and approximately 6-fold, respectively, than those of the control mice (Figure 1).

Although the above results indicated that the 5-week-old *db/db* mice did not have type 2 diabetes, the 12-week-old mice had developed type 2 diabetes with insulin resistance and obesity.

### 3.2. Dermal Water Content and Skin Tissue Findings

**Figure 2:**
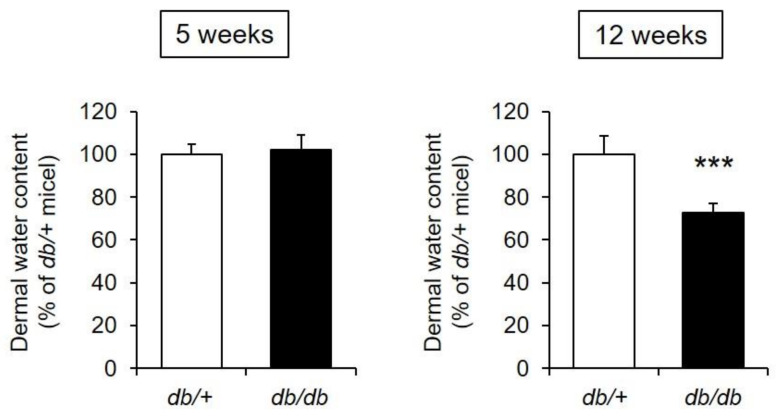
Dermal water content. The dermal water content was measured in *db/+* and *db/db* mice at 5 and 12 weeks of age (mean ± S.D., *n* = 5, ***: *p* < 0.001 vs. *db*/+ mice).

The dermal water content of the 5-week-old *db/db* mice was almost the same as that of the control mice. In contrast, the dermal water content of the 12-week-old *db/db* mice was decreased by approximately 70% compared with that of the control mice, and this difference was significant (Figure 2).

From the above, the 12-week-old *db/db* mice were determined to have xeroderma similar to that found in diabetic patients [2,3,4].

### 3.3. mRNA Expression Levels of Functional Genes in the Skin

The water content of the skin is maintained by various factors, such as filaggrin, loricrin, ceramide, hyaluronic acid, and collagen [13,14,15]. Therefore, expression levels of the following skin functional genes were measured in the skin of *db/db* mice: filaggrin, loricrin, ceramide synthases (serine palmitoyltransferase 1; Sptlc1, serine palmitoyltransferase 2; Sptlc2), ceramide-degrading enzymes (alkaline ceramidase 1; Acer1, N-acylsphingosine amidohydrolase 1; Asah1), hyaluronic acid synthase 2 (Has2), a hyaluronic acid-degrading enzyme (hyaluronidase 1; Hyal1), and type I collagen (collagen type I alpha 1 chain; Col1a1, collagen type I alpha 2 chain; Col1a2).

**Figure 3:**
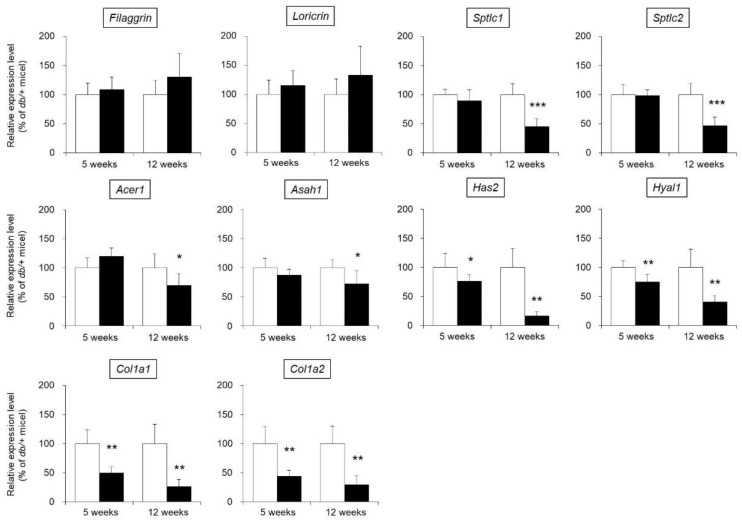
mRNA expression levels of skin functional genes. The skin of 5-week-old and 12-week-old *db/+* (white) and *db/db* mice (black) was removed, and the mRNA expression levels of filaggrin, loricrin, Sptlc1, Sptlc2, Acer1, Asah1, Has2, Hyal1, Col1a1, and Col1a2 were measured by real-time PCR. These genes were normalized to β-actin, and the mean value in *db/+* mice was set to 100% (mean ± SD, *n* = 5, *: *p* < 0.05, **: *p* < 0.01, ***: *p* < 0.001 vs. *db/+* mice).

No difference in the mRNA expression levels of filaggrin or loricrin in the skin were observed between *db/db* mice and the control mice. The expression levels of Sptlc1, Sptlc2, Acer1, and Asah1 were not significantly different in the 5-week-old *db/db* and control mice but were significantly decreased in the 12-week-old *db/db* mice compared with the control mice. The expression levels of Has2, Hyal1, Col1a1, and Col1a2 were significantly lower in the *db/db* mice than in the control mice at both 5 and 12 weeks of age (Figure 3).

### 3.4. AQP Expression in the Skin

**Figure 4:**
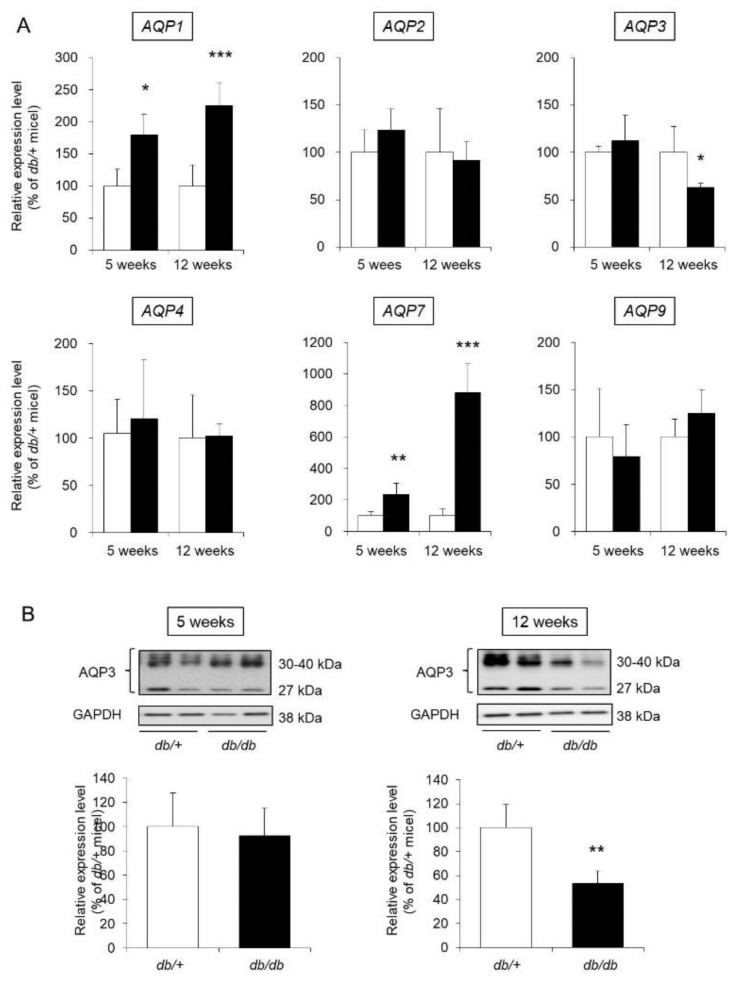
AQP expression in the skin. The skin of 5-week-old and 12-week-old *db/+* (white) and *db/db* mice (black) was removed. (**A**) The mRNA expression levels of AQP were measured by real-time RT-PCR, and after normalization to β-actin, the average value in *db/+* mice was set to 100%. (**B**) AQP3 protein expression levels were analyzed by western blotting, and after normalization to GAPDH, the average value in *db/+* mice was set to 100% (mean ± SD, *n* = 5, *: *p* < 0.05, **: *p* < 0.01, ***: *p* < 0.001 vs. *db/+* mice).

Thirteen AQP species (from AQP0 to AQP12) are widely distributed throughout the body in mammals [16]. Among the analyzed AQPs, the expression of AQP1, AQP2, AQP3, AQP4, AQP7, and AQP9 in mouse skin was confirmed in this study. Regarding the mRNA expression levels of AQP2, AQP4, and AQP9, no difference was observed between *db/db* mice and control mice at either 5 weeks old or 12 weeks of age. The expression levels of AQP1 and AQP7 were significantly higher in the *db/db* mice than in the control mice at both 5 and 12 weeks of age. The expression level of AQP3 was not significantly different in 5-week-old *db/db* and control mice but was significantly lower (decreased by approximately 50%) in 12-week-old *db/db* mice than in 12-week-old control mice (Figure 4A).

AQP3, the most widely expressed AQP in skin, is closely related to retention of the dermal water content [17]. Therefore, we focused on AQP3 and analyzed its protein expression level. When the protein expression level of AQP3 was analyzed by western blotting, AQP3 bands at approximately 27 kDa and 30–40 kDa were detected. It has been reported that these molecular weights correspond to nonglycosylated AQP3 (27 kDa) and glycosylated AQP3 (30–40 kDa) [18,19]. Although the stability and translocation of AQP3 to the cell membrane differ with and without glycosylation, the presence or absence of glycosylation does not affect the water permeation function of AQP3 [20,21,22]. In this study, the sum of the signals from these bands was analyzed and used to determine the expression level of AQP3. As a result, the protein expression level of AQP3 in 5-week-old *db/db* mice was similar to that in 5-week-old control mice. In contrast, the expression level of AQP3 in 12-week-old *db/db* mice was significantly lower than that in 12-week-old control mice (Figure 4B).

From the above results, changes throughout the development of diabetes were found to be dependent on AQP type. Furthermore, the expression level of AQP3 in the skin was revealed to be decreased at both the mRNA and protein levels with the progression of type 2 diabetes.

### 3.5. Effect of Glucose on AQP3 Expression in HaCaT Cells

**Figure 5:**
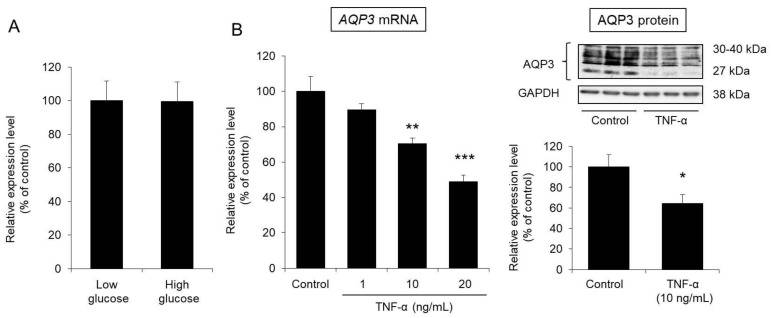
Effect of glucose and TNF-α on AQP3 expression in HaCaT cells. (**A**) HaCaT cells were cultured for 24 h in DMEM containing glucose at a low or high concentration. Total RNA was extracted from the cells, and the mRNA expression level of AQP3 was analyzed by real-time RT-PCR. After normalization to GAPDH, the average expression level in the control (low glucose) was set to 100%. (**B**) TNF-α was added to HaCaT cells, which were then cultured for 24 h. Total RNA was extracted from the cells, and the mRNA expression level of AQP3 was analyzed by real-time RT-PCR. The protein expression level of AQP3 was analyzed by western blotting. All data were normalized to GAPDH and are expressed with the average value of the control group set to 100% (mean ± S.D., *n* = 4, *: *p* < 0.05, **: *p* < 0.01, ***: *p* < 0.001 vs. control).

An in vitro experiment was carried out to examine whether the decreased expression of cutaneous AQP3 observed in diabetes is caused by an increase in blood glucose levels. In particular, we analyzed the expression level of AQP3 in human HaCaT epidermal keratinocyte cells under high-glucose (4500 mg/L) and low-glucose (1000 mg/L) conditions. Even when glucose at a high concentration was added to the HaCaT cells, the mRNA expression level of AQP3 was almost the same as that measured when glucose was added at a low concentration (Figure 5A).

From the above, glucose is clearly unlikely to directly reduce the expression level of AQP3 in the skin.

### 3.6. Effect of TNF-α on AQP3 Expression in HaCaT Cells

TNF-α, an inflammatory cytokine, was reported to decrease the expression level of AQP3 [10]. Therefore, we examined whether TNF-α would decrease the expression level of AQP3 in HaCaT cells.

After TNF-α was added to HaCaT cells and the cells were cultured for 24 h, the mRNA expression level of AQP3 was significantly decreased in a concentration-dependent manner compared with that of the control cells. The protein expression level of AQP3 was also significantly decreased to approximately 60% of that in the control cells due to the addition of TNF-α (Figure 5B).

From the above, TNF-α was confirmed to decrease the expression level of AQP3 in epidermal keratinocytes, as found in a previous report [10].

### 3.7. Blood TNF-α Concentration

TNF-α decreases the expression level of AQP3 in epidermal keratinocytes. Therefore, we next investigated the induction of systemic inflammation in 12-week-old *db/db* mice by measuring blood TNF-α concentration.

**Figure 6:**
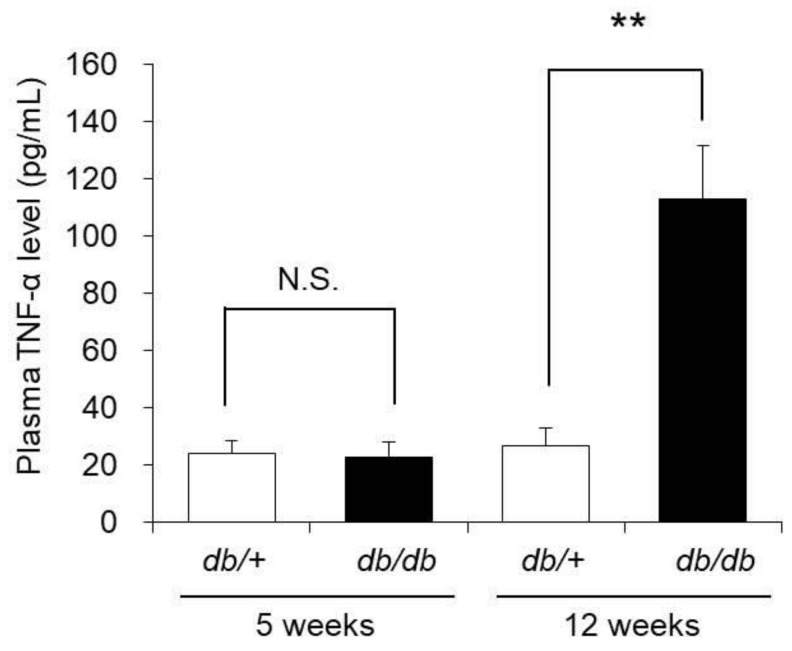
Blood TNF-α concentration. The blood TNF-α concentrations of 5-week-old and 12-week-old *db/+* and *db/db* mice were measured with ELISA kits (mean ± S.D., *n* = 5, N.S.: not significant, **: *p* < 0.01).

The blood TNF-α concentration in 5-week-old *db/db* mice was almost the same as that in 5-week-old control mice. In contrast, the blood TNF-α concentration in 12-week-old *db/db* mice was approximately 4-fold higher than that in the 12-week-old control mice (Figure 6).

From the above, systemic inflammation was revealed to be induced in 12-week-old *db/db* mice in which cutaneous AQP3 expression was decreased.

### 3.8. mRNA Expression Levels of TNF-α, iNOS, and COX-2 in the Skin

We investigated the presence of local inflammation in the skin of *db/db* mice. In particular, the mRNA expression levels of TNF-α, inducible nitric oxide synthase (iNOS), and cyclooxygenase-2 (COX-2), whose expression is increased due to inflammation [23,24], were evaluated as indices of inflammation.

**Figure 7:**
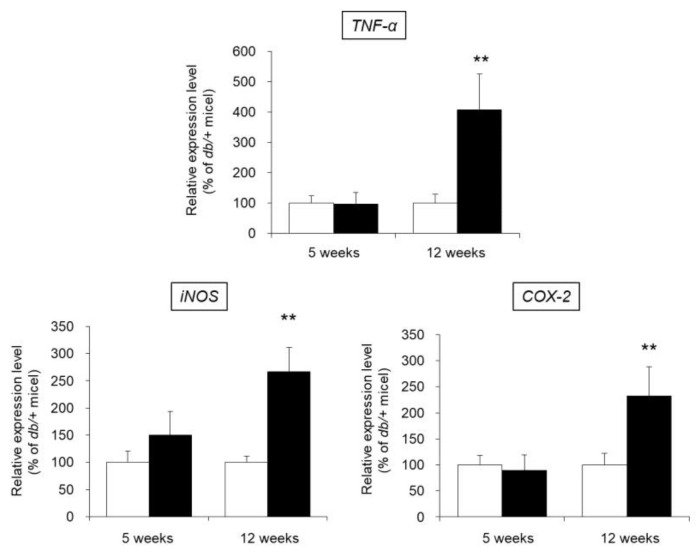
mRNA expression levels of TNF-α, iNOS, and COX-2 in the skin. The skin of 5-week-old and 12-week-old *db/+* (white) and *db/db* mice (black) was removed, and the mRNA expression levels of TNF-α, iNOS, and COX-2 were measured by real-time RT-PCR. These values were normalized to β-actin, and the mean value in the *db/+* mice was set to 100% (mean ± SD, *n* = 5, **: *p* < 0.01 vs. *db/+* mice).

The mRNA expression level of TNF-α in the skin of 5-week-old *db/db* mice was almost the same as that of 5-week-old control mice. In contrast, the expression level of TNF-α was significantly higher (increased by approximately 4-fold) in 12-week-old *db/db* mice than in 12-week-old control mice. Similarly, the expression levels of iNOS and COX-2 were no different in the 5-week-old *db/db* and control mice, whereas these levels were significantly higher in 12-week-old *db/db* mice compared to 12-week-old control mice (Figure 7).

The above results show that in 12-week-old *db/db* mice in which decreased cutaneous AQP3 expression was observed, local inflammation was induced in the skin.

## 4. Discussion

Many patients with diabetes develop xerosis, but many details of the mechanism are unclear. We previously found that the expression level of AQP3 in the skin of model mice with STZ-induced type 1 diabetes was significantly decreased when the skin was dry [8]. In this study, to investigate diabetic xerosis overall, we used model *db/db* mice with type 2 diabetes.

When the blood glucose and blood insulin levels of the *db/db* mice were measured, both were similar in 5-week-old *db/db* and control mice, whereas in 12-week-old mice, both the blood glucose level and blood insulin level were significantly increased in the *db/db* mice compared to the control mice. Therefore, the development of diabetes with insulin resistance was confirmed (Figure 1). The dermal water content of the *db/db* mice was almost the same as that of control mice at 5 weeks of age but significantly decreased at 12 weeks of age (Figure 2). From the above, dryness of the skin was observed in *db/db* mice that presented with the pathology of type 2 diabetes, and this phenomenon was similar to clinical reports that diabetic patients develop xeroderma [3,4].

We investigated whether dryness of the skin during type 2 diabetes is related to the altered expression of AQP3, which plays an important role in maintaining the dermal water content [7]. The expression level of skin AQP3 in 5-week-old *db/db* mice was almost the same as that in control mice of the same age, but skin AQP3 expression was significantly decreased in the *db/db* mice compared to the control mice at 12 weeks of age (Figure 4). In addition, the decrease in AQP3, increase in blood glucose level, and decrease in dermal water content were correlated. The dermal water content was reported to be decreased in AQP3-knockout mice [7]. In addition, cutaneous AQP3 is known to change in various skin disorders [25]; for example, AQP3 is decreased in psoriasis [26] and vitiligo [27,28] and during aging [29], causing skin dryness. Furthermore, it was recently found that a decrease in skin AQP3 was involved in the dry skin observed upon treatment with an epidermal growth factor receptor (EGFR) inhibitor [30]. Therefore, xeroderma during type 2 diabetes may occur as a result of a decrease in cutaneous AQP3, which limits the movement of water from the vascular side of the skin to the stratum corneum, as observed in type 1 diabetes [8]. The dermal water content is maintained by various factors other than AQPs, and if the balance in these factors is broken, the dermal water content will decrease [13,14,15]. By analyzing the expression levels of various skin functional genes, the ceramide synthase genes Sptlc1 and Sptlc2 and ceramide-degrading enzymes Acer1 and Asah1 were found to be significantly changed at only 12 weeks old of age (Figure 3). While the amount of skin ceramide was not measured, the amount of skin ceramide in the 12-week-old *db/db* mice is thought to have been unchanged compared to that of the control mice since the level of all of the major enzymes responsible for ceramide synthesis and degradation were decreased. Therefore, a decrease in AQP3 is more important in the development of diabetic xerosis than a decrease in the amount of ceramide.

Why did the expression level of cutaneous AQP3 decrease during type 2 diabetes? First, we investigated whether an increase in blood glucose levels had been involved in the decrease in cutaneous AQP3 using HaCaT cells. When HaCaT cells were treated with a high concentration of glucose, the expression level of AQP3 was not changed (Figure 5A). Therefore, it is unlikely that the decreased expression of cutaneous AQP3 observed during diabetes is directly related to an increase in blood glucose levels.

Patients with type 2 diabetes often suffer from obesity, which causes persistent systemic inflammation and is considered one of the causes of diabetic complications [9]. Additionally, inflammatory cytokines such as TNF-α decreased the expression level of AQP3 in various cell types [10,31]. In this study, the addition of TNF-α to HaCaT cells significantly decreased the expression level of AQP3 (Figure 5B). Therefore, we focused on inflammation as the mechanism of the decrease in AQP3 in type 2 diabetes and performed an analysis. No change in blood TNF-α concentration or skin TNF-α, iNOS, and COX-2 mRNA expression levels was observed in 5-week-old *db/db* mice, which did not show a decrease in skin AQP3. In contrast, all of these parameters were significantly higher in 12-week-old *db/db* mice, in which decreased AQP3 was observed (Figs. 6 and 7). In addition, these inflammatory markers and decreased AQP3 were correlated. Therefore, systemic and local inflammation in the skin may occur during type 2 diabetes, which may decrease AQP3. In model mice with STZ-induced type 1 diabetes, in which a decrease in skin AQP3 was observed, both the blood TNF-α concentration and cutaneous TNF-α expression were increased (Figure S1). Therefore, the decrease in cutaneous AQP3 observed in the mice may also have been triggered by inflammation.

AQP3 is distributed in not only the skin but also the large intestine [18,32] and kidney [33,34]. When the expression levels of AQP3 in these organs were analyzed in 12-week-old *db/db* mice, a significant decrease in AQP3 was observed in the colon, but AQP3 expression was significantly increased in the kidney (Figure S2). The AQP3 expression level in the colon is significantly decreased with inflammation [10,31], consistent with the results of this study. On the other hand, the expression level of AQP3 in the kidney is strongly regulated by the antidiuretic hormone vasopressin, and it is known that vasopressin secretion is enhanced and renal AQP3 is increased during diabetes [35]. The results of this study strongly suggest that the regulation of AQP3 expression in the kidney during diabetes depends on vasopressin rather than inflammatory cytokines.

In this study, the expression of AQP1, AQP2, AQP4, AQP7, and AQP9 in mouse skin was confirmed, and AQP3, AQP1, and AQP7 levels were significantly higher in 12-week-old *db/db* mice than in control mice (Figure 4A). AQP1 is known to be expressed in melanocytes involved in melanin synthesis [36]. AQP7 is expressed in adipocytes and is involved in the transport of glycerol and water [37], similar to the function of AQP3. However, many details regarding the functions of AQP1 and AQP7 in the skin remain unclear. By clarifying the functions of these AQPs, the significance of their increased expression in type 2 diabetes will become clear in the future.

Delayed wound healing accompanied by a reduction in the dermal water content has been reported during diabetes [38,39]. Cutaneous AQP3 is involved in cell proliferation and cell migration [40,41], and delayed wound healing has been confirmed in AQP3-deficient mice [7]. It has been reported that AQP3 in the skin is decreased in STZ-induced type 1 diabetic model rats, which may cause delayed wound healing in diabetes [42]. In addition, it was clarified that the skin of pigs with experimentally induced type 1 diabetes has a lower AQP3 compared to non-diabetic pigs, that wound healing is delayed in diabetes model pigs, and that topical treatment of erythropoietin accelerates wound healing and this effect depends on the increase of AQP3 [43]. Therefore, a decrease in cutaneous AQP3 may be involved in not only xerosis but also delayed wound healing during diabetes.

The results of this study revealed that cutaneous AQP3 decreased at the onset of diabetic xerosis, which may have been triggered by inflammation. Therefore, these results suggest that not only substances that directly increase AQP3 in the skin but also anti-inflammatory drugs and anti-TNF-α antibody would be effective for the treatment and prevention of diabetic xerosis. In fact, curcumin was found to suppress the onset of skin disorders during diabetes, suggesting that this may be due to its anti-inflammatory effect [44]. A peroxisome proliferator-activated receptor γ (PPARγ) agonist used as an insulin secretagogue was found to show anti-inflammatory effects in addition to its effect in increasing AQP3 [45,46,47,48,49] and may be useful for diabetic xerosis. In the future, the application of these drugs in diabetic xerosis is expected.

As mentioned above, it has been reported that cutaneous AQP3 decreases during psoriasis [26]. On the other hand, metabolic syndrome including type 2 diabetes is associated with an increased risk of psoriasis [50]. From these facts, it is considered that the decrease in cutaneous AQP3 during type 2 diabetes may contribute to the increased risk of psoriasis. Therefore, the results of this research may affect various other dermatological conditions, and future development is expected.

## 5. Conclusions

In summary, xeroderma during type 2 diabetes may occur as a result of a decrease in cutaneous AQP3 as observed in type 1 diabetes. Therefore, a substance that increases AQP3 may be useful for improving xeroderma. Additionally, a decrease in skin AQP3 may be triggered by inflammation. Therefore, anti-inflammatory drugs may be effective as new therapeutic agents for diabetic xerosis.

## Data Availability

Not applicable.

## Supplementary Materials

The following are available online at https://www.mdpi.com/2227-9059/9/2/104/s1, Table S1: mRNA primer sequences, Figure S1: Inflammatory markers in STZ-induced type 1 diabetes model mice, Figure S2: AQP3 mRNA expression levels in the colon and kidney.
