# Supplementary material for: Role of Cutaneous Aquaporins in the Development of Xeroderma in Type 2 Diabetes
Source: Biomedicines. 2021 Jan 21;9(2):104. doi: 10.3390/biomedicines9020104 (PMC7912687; doi:10.3390/biomedicines9020104)
Supplement: Supplementary file 1 [file biomedicines-09-00104-s001.pdf]

Supplementary Table 1 mRNA primer sequences.

Gene	Forward	Reverse
mAQP0	TGCTCTGCATCTTTGCTACA	GCACCAGTGTAATACATCCCA
mAQP1	CTGCTGGCGATTGACTACACT	TCATAGATGAGCACTGCCAGG
mAQP2	CTGGCTGTCAATGCTCTCCAC	TTGTCACTGCGGCGCTCATC
mAQP3	CCTTGTGATGTTTGGCTGTGG	GGAAGCACATTGCGAAGGTC
mAQP4	GAGTCACCACGGTTCATGGA	CGTTTGGAATCACAGCTGGC
mAQP5	GCTGGAGAGGCAGCATTG	CACCCAAGTGTCCCATCATG
mAQP7	GCTTGGTCTGCTGCTTCAG	GGAACTCTGCCAGAAACTCTC
mAQP8	GGCTTCTCTGTCATTGTGGA	TCCGATGAGGAGCCTAATGA
mAQP9	TGAGCCATTAGGAGAGACCTT	ACCTCCAACTTTAGTCCACCA
mFilaggrin	AAGGAAATCAGTCTTGCCGT	CTGACCTTCTGAGACACACC
mLoricrin	GCCGATGGGCTTAACTTTCT	CAGGATACACCTTGAGCGAC
mAcer1	CCGAGTTCTACAATACGTTCA	CATACGGATGCATGAGGAAC
mAsah1	CTGTCCTCAACAAGCTGACTG	TCTCAGTACGTCCTCAAGGC
mSptlc1	TCCCCTTCCAGAACTGGTTAAA	CCATAGTGCTCGGTGACT
mSptlc2	GTCAGGAAATTGGAAACCTGG	AGCTTCCACACCTAAGAACC
mHyal1	TTTCTTTGAGCCTGGAGCTA	GTAGTTTCCTTTCGTTGGCT
mHas2	CGTGGATTATGTACAGGTGTGT	CCAACACCTCCAACCATAGG
mCol1a1	CCCGAGGTATGCTTGATCTG	GGTGATACGTATTCTTCCGGG
mCol1a2	TCTCACTCCTGAAGGCTCTA	GTAGTAATCGCTGTTCCACTC
mTNF-α	ATGGACACCAAACATTTCCTGC	CCAGTGGAGAGCCGATTCC
mCOX-2	CAGGGCCCTTCCTCCCGTAG	GCCTTGGGGGTCAGGGATGA
miNOS	GGCAGCCTGTGAGACCTTTG	GCATTGGAAGTGAAGCGTTTC
mβ-actin	GAGCGCAAGTACTCTGTGTG	CGGACTCATCGTACTCCTG
hAQP3	AGACAGCCCCTTCAGGATTT	TCCCTTGCCCTGAATATCTG
hGAPDH	GGCAAATTCAACGGCACAGT	AGATGGTGATGGGCTTCCC

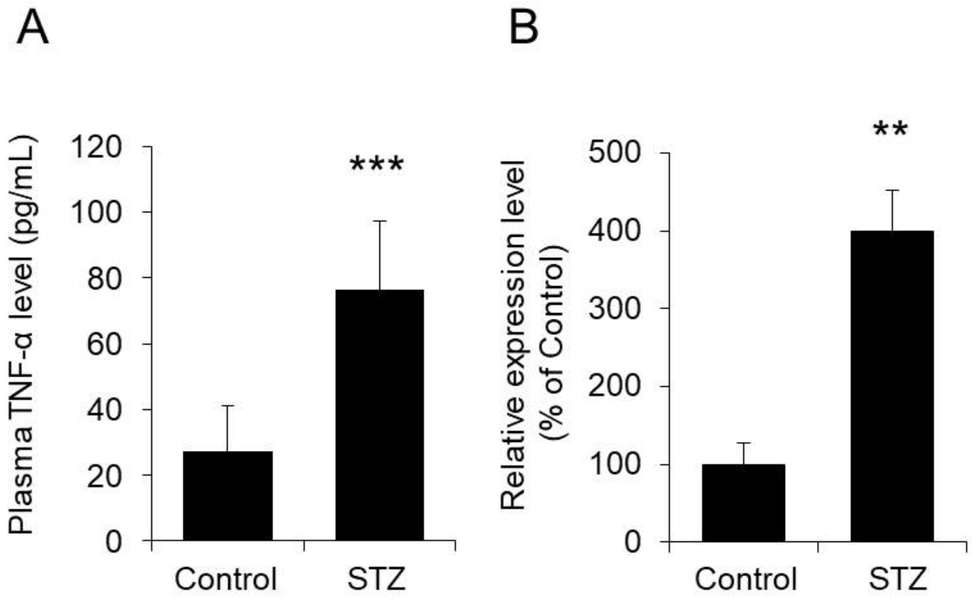

Figure S1. Inflammatory markers in STZ-induced type 1 diabetes model mice. STZ (150 mg/kg) was intravenously administered to mice by the tail vein, and after breeding for 4 weeks, the TNF-α concentration in the blood was measured by ELISA (A). After the skin had been removed, the mRNA expression level of TNF-α was measured by real-time RT-PCR, and the mean value in the control group after correction with GAPDH was set to 100% (B). Mean ± S.D., n=5, **; $p<0.01$, ***; $p<0.001$ vs. control mice.

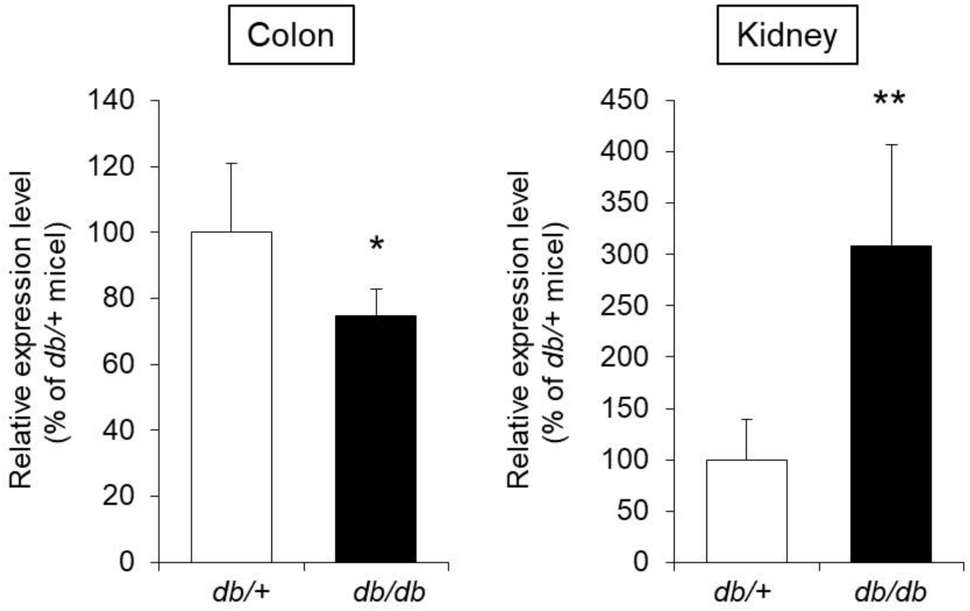

Figure S2. AQP3 mRNA expression levels in the colon and kidney. The colon and kidney were removed from 5-week-old *db*/+ and *db*/*db* mice, and the mRNA expression levels of AQP3 were measured by real-time RT-PCR. After correction with GAPDH, the average value in the *db*/+ mice was set to 100% (mean±SD, n=5, *; $p<0.05$, **; $p<0.01$ vs *db*/+ mice).
